# Surgical Site Infection by Shewanella algae After Major Surgery in a Patient With Colorectal Cancer: Case Report

**DOI:** 10.7759/cureus.23158

**Published:** 2022-03-14

**Authors:** Cristel M Rodriguez-Vargas, Monica R Pachar-Flores, Irene Espinosa, Eliecer Cherigo

**Affiliations:** 1 Internal Medicine, Hospital Santo Tomas, Panama, PAN; 2 Infectious Diseases, Instituto Oncologico Nacional, Panama, PAN; 3 Infectious Diseases, Hospital Santo Tomás, Panama, PAN; 4 Microbiology Laboratory, Instituto Oncologico Nacional, Panama, PAN

**Keywords:** sea bacteria, bacterial infection, infectious disease, surgical wound infection, shewanella algae

## Abstract

Surgical site infections are a common complication in the management of patients with solid cancer tumors in cytotoxic chemotherapy. The antibiotic combination chosen depends on the neoadjuvant therapy used and the anatomical site and extent of the surgery. In this brief report, we describe a surgical site infection caused by an unusual microorganism and a succinct review of the pathogen's behavior.

## Introduction

*Shewanella* species are a group of bacteria commonly found in aquatic habitats. The first description of the species was in 1931 from water supplies of dairies. First known as *Achromobacter putrefaciens*, then* Pseudomonas*, in 1985, they were finally reclassified as a new genus, *Shewanella* [1]. A variety of infections caused by this bacteria have been reported in humans, mostly associated with water exposure and immunosuppression. Some reports include skin and soft tissue infections, otitis externa, bacteremia, and others [2].

Surgical site infections are an unexpected and unique presentation of Shewanella sp, with only few previous case reports, associated with *Shewanella putrefaciens*. Here, we present the first *Shewanella algae* surgical site infection, without previous common exposures or nosocomial outbreak association. 

## Case presentation

A 74-year-old woman with sigmoid cancer, adenocarcinoma (T3N0M0), was admitted to our hospital with acute abdominal pain. The patient had a recent diagnosis of colorectal cancer and went to a scheduled surgery during the first week of December. She has a previous history of diabetes, hypertension, and fibrocystic breast disease. She is from an urban location in Panama City with no history of travel due to the COVID-19 pandemic and a recent diagnosis of cancer. She has no previous exposure to seawater or any marine environment exposure, neither a history of eating seafood nor fish.

After careful preoperative assessment and control of her comorbidities, the patient underwent a scheduled lower anterior resection with colorectal anastomosis without immediate complications. On the seventh postoperative (PO) day, she returned to the emergency room with a 48-hour history of nausea, vomiting, abdominal distention, and pain. An emergency exploratory laparotomy was performed, where they found a 50% dehiscence of the colorectal anastomosis, together with fecal material that drained through the intestine into the pelvic cavity, with inflammatory material. Surgical dismantling of the colorectal anastomosis with terminal colostomy and cavity wash were done.

**Figure 1:**
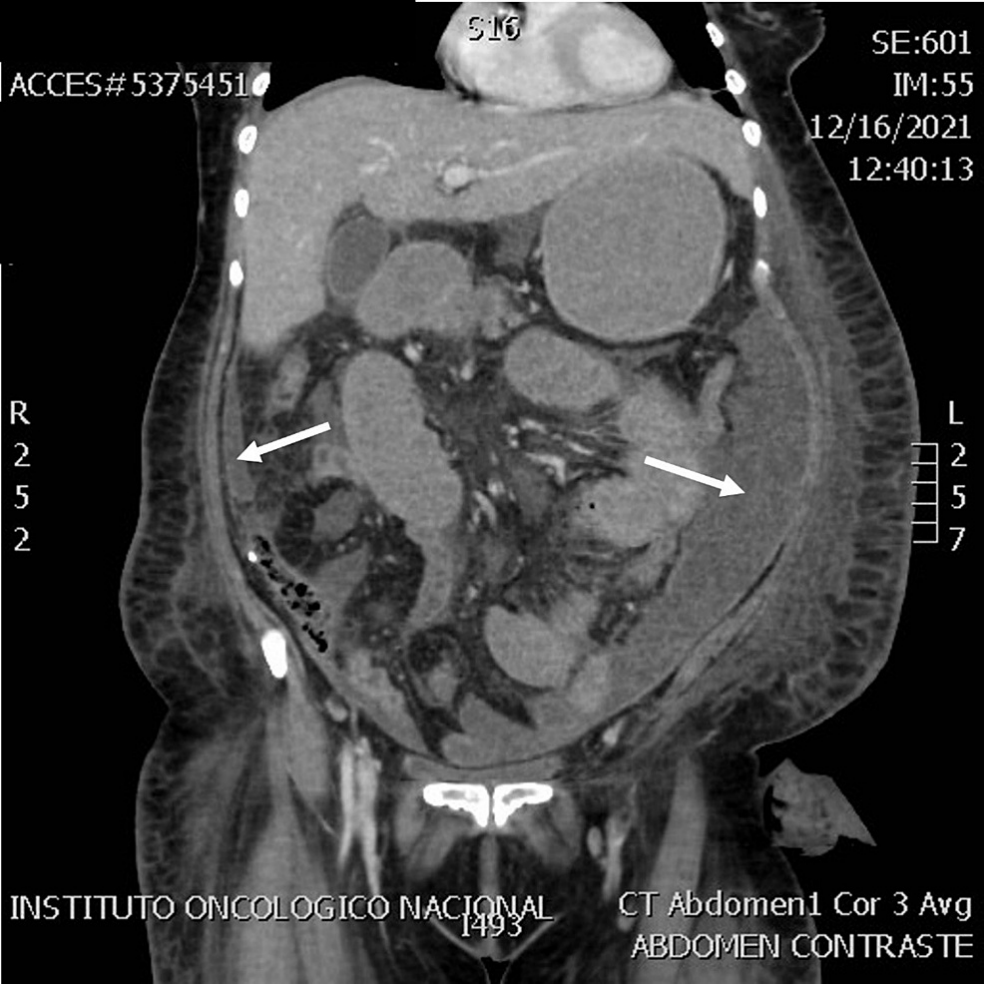
Intra-abdominal collection associated with positive culture for Shewanella algae White arrows show right and left paracolic gutter collections.

**Figure 2:**
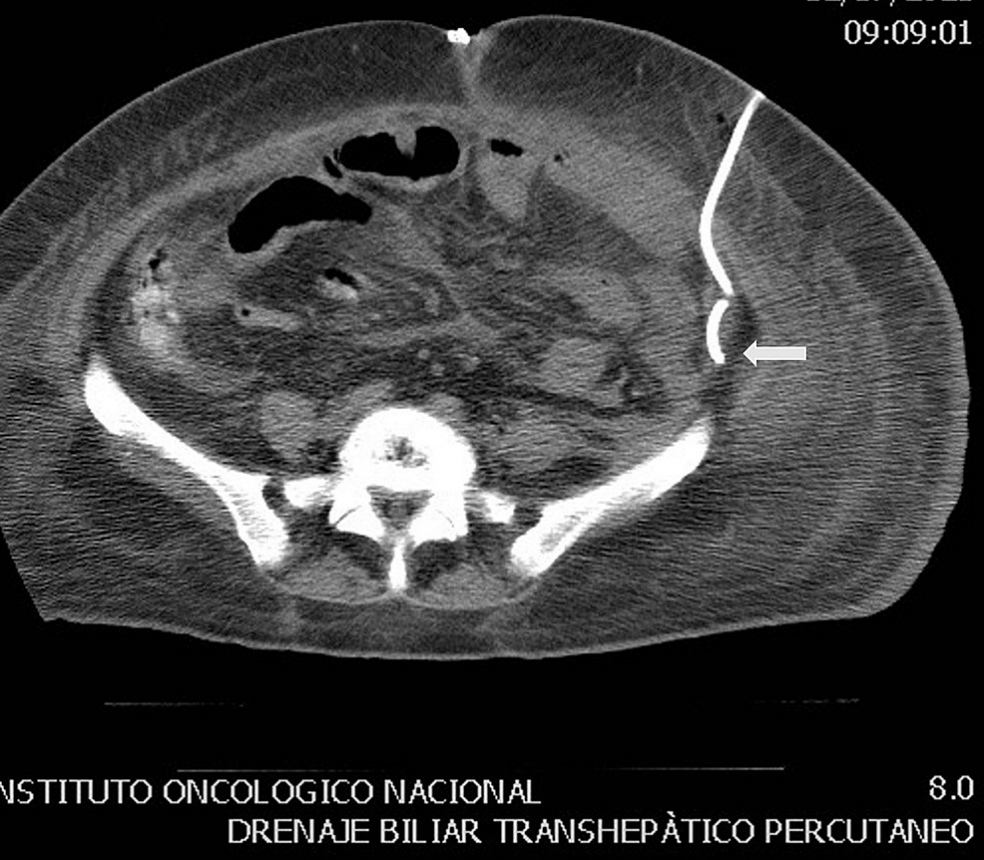
Percutaneous drainage of intra-abdominal collection The image above shows percutaneous drainage of the left paracolic gutter collection (700 ml) (white arrow).

She was admitted to the intensive care unit (ICU) where she had a torpid evolution with septic shock. Cefepime and metronidazole were initiated. During the fifth day after admission, she developed colostomy necrosis and was taken again to the operating room (OR) for colostomy revision where a parastomal abscess was found. After this, the patient remained stable until the 15th day PO, when the systemic inflammatory response syndrome (SIRS) signs appeared again. Space surgical site infection was suspected, and an abdominal computed tomography (CT) scan was done, which revealed intra-abdominal collections in the left paracolic gutter, right paracolic gutter, and pelvic cavity next to the rectal stump. The left paracolic gutter collection was drained percutaneously, and 700 ml of pus was collected (Figures 1, 2). The pus was cultured for bacteria, mycobacteria, and fungi resulting positive for *Shewanella algae *and *Enterococcus faecalis,* using matrix-assisted laser desorption/ionization and time-of-flight mass spectrometry (MALDI-TOF MS).

Antibiotic regimen was then switched to intravenous imipenem 500 mg twice a day (q12h) and vancomycin 1 g q12h (ampicillin was not available at the time). Due to the extremely unusual isolation of this bacteria and the implications in nosocomial outbreaks, the patient went under contact isolation precautions; the sterile solutions used for abdominal cavity wash were analyzed and colonization screening was implemented to the operating room staff, nonetheless the results were negative. Several hospitalized patients were tested for colonization, with negative results. 

Besides the antibiotic and surgical therapy, the wound was managed with vacuum-assisted closure (VAC) and finally resolved at the 25th PO. A culture was taken from the fluid of the VAC sponge on the 23rd PO which was positive for *Shewanella algae. *The patient had a favorable clinical course and, after one month of hospitalization, was discharged. On the follow-up appointment, two weeks after the hospital discharge, she was afebrile with a functioning colostomy and had scheduled chemotherapy treatment plan. This is the first *Shewanella algae* associated surgical site infection reported in Panama, and the second *Shewanella* species associated surgical site infection case published in the English literature.

## Discussion

*Shewanella* species are Gram-negative, nonfermentative, motile bacilli. They are widely distributed in the environment and have uncommonly been associated with a variety of human infections [2]. They were first isolated from dairy products in 1931; nowadays, the most common type of association is marine environments and is sporadically reported in association with soil, fish, meat, natural gas, and petroleum reserves [3]. Although the incidence of *Shewanella* disease in humans remains low compared with other pathogens, there are an increasing number of reports, probably because of better diagnostic tools.

*Shewanella putrefaciens* and *Shewanella algae* are the species often associated with human infections; however, *S. haliotis* and *S. xiamenensis* have also been reported [2]. Automated identification systems lack the capacity to identify between *S. putrefaciens *and *S. algae* because the latter species is not included in many databases [3,4]. In our case, we were able to obtain a positive result of* S. algae *from an abdominal fluid aspirate and later from a surgical sponge, thanks to matrix-assisted laser desorption/ionization time-of-flight mass spectrometry (MALDI-TOF MS).

This strange organism has been associated with otitis externa, soft tissue infections, bacteremia, hepatobiliary infections, endocarditis, pneumonia, cerebral abscess, and meningoencephalitis [3-6]. The most extensive compilation of *Shewanella* sp human infections was made in 2013, which reported 239 cases, none of them were surgical site infections [7]. To our knowledge, there is only one case report of surgical site infection associated with *Shewanella putrefaciens* after abdominal hernioplasty [8]. There have been* Shewanella* species isolations from intra-abdominal sources, most of them are thought to be colonization [9].

The major risk factors for *Shewanella* infections are contact with seawater with a previously injured skin via recreational or occupational exposure and consumption of seafood (70% of cases) [3]. In a recent review in Spain, unambiguous exposure to water was reported in the minority of cases and nosocomial infection was suspected [4]. Conditions affecting the immune response such as malnutrition, anemia, renal failure, diabetes, cancer, and liver cirrhosis seem to be more predisposed to *Shewanella* infections [10]. In our case, the patient did not have exposure to marine environment or seafood or fish but was immunocompromised, which is a risk factor for unusual infections. Investigations were made to exclude nosocomial infection, including colonization screening for the healthcare staff and culture of the sterile solutions used for abdominal wash during surgery, with negative results. *Shewanella algae* is susceptible to aminoglycosides, carbapenems, erythromycin, and quinolones [11]. Many *Shewanella* species are susceptible to narrower spectrum antibiotics such as amoxicillin and third-generation cephalosporins; however, they can show resistance to imipenem by secreting oxacillinases [7]. Our patient was treated successfully with imipenem, with a final good outcome.

## Conclusions

*Shewanella *species, although an infrequent cause of infections, appear to have an increasing number of outbreaks and reports. Regarding surgical site infections, *Shewanella* is an extremely rare association, probably because the common entry mechanism is through injured skin in contact with water, fish or seafood consumption, or medical equipment contamination. It is important to count on new generation techniques such as MALDI-TOF MS to make the accurate identification with susceptibility to guide antibiotic therapy.
